# Adjuvant Transthoracic Negative-Pressure Ventilation in Nonintubated Thoracoscopic Surgery

**DOI:** 10.3390/jcm12134234

**Published:** 2023-06-23

**Authors:** Riccardo Taje, Eleonora Fabbi, Roberto Sorge, Stefano Elia, Mario Dauri, Eugenio Pompeo

**Affiliations:** 1 Department of Thoracic Surgery, Policlinico Tor Vergata University, V.le Oxford 81, 00133 Rome, Italy; r-taje@virgilio.it; 2 Department of Anesthesia and Intensive Care, Policlinico Tor Vergata University, V.le Oxford 81, 00133 Rome, Italy; eleonora.fabbi@ptvonline.it (E.F.); mario.dauri@ptvonline.it (M.D.); 3 Department of Biostatistics, Tor Vergata University of Rome, 00133 Rome, Italy; sorge@uniroma2.it; 4 Department of Medicine and Health Sciences V. Tiberio, University of Molise, 86100 Campobasso, Italy; stefano.elia@unimol.it

**Keywords:** nonintubated thoracic surgery, VATS, spontaneous ventilation, emphysema, interstitial lung disease

## Abstract

Background: To minimize the risks of barotrauma during nonintubated thoracoscopic-surgery under spontaneous ventilation, we investigated an adjuvant transthoracic negative-pressure ventilation (NPV) method in patients operated on due to severe emphysema or interstitial lung disease. Methods: In this retrospective study, NPV was employed for temporary low oxygen saturation and to achieve end-operative lung re-expansion during nonintubated lung volume reduction surgery (LVRS) for severe emphysema (30 patients, LVRS group) and in the nonintubated wedge resection of undetermined interstitial lung disease (30 patients, wedge-group). The results were compared following 1:1 propensity score matching with equivalent control groups undergoing the same procedures under spontaneous ventilation, with adjuvant positive-pressure ventilation (PPV) performed on-demand through the laryngeal mask. The primary outcomes were changes (preoperative–postoperative value) in the arterial oxygen tension/fraction of the inspired oxygen ratio (ΔPO_2_/FiO_2_;) and ΔPaCO_2_, and lung expansion completeness on a 24 h postoperative chest radiograph (CXR-score, 2: full or 1: incomplete). Results: Intergroup comparisons (NPV vs. PPV) showed no differences in demographic and pulmonary function. NPV could be accomplished in all instances with no conversion to general anesthesia with intubation. In the LVRS group, NPV improved ΔPO_2_/FiO_2_ (9.3 ± 16 vs. 25.3 ± 30.5, *p* = 0.027) and ΔPaCO_2_ (−2.2 ± 3.15 mmHg vs. 0.03 ± 0.18 mmHg, *p* = 0.008) with no difference in the CXR score, whereas in the wedge group, both ΔPO_2_/FiO_2_ (3.1 ± 8.2 vs. 9.9 ± 13.8, *p* = 0.035) and the CXR score (1.9 ± 0.3 vs. 1.6 ± 0.5, *p* = 0.04) were better in the NPV subgroup. There was no mortality and no intergroup difference in morbidity. Conclusions: In this retrospective study, NITS with adjuvant transthoracic NPV resulted in better 24 h oxygenation measures than PPV in both the LVRS and wedge groups, and in better lung expansion according to the CXR score in the wedge group.

## 1. Introduction

Nonintubated thoracic surgery, entailing thoracoscopic surgery under spontaneous ventilation without tracheal intubation, has shown reduced morbidity and hospital stays compared to intubated thoracic surgery, entailing oro-tracheal intubation and single-lung mechanical ventilation [1,2]. Moreover, in patients with impaired pulmonary function [3], nonintubated thoracic surgery resulted in better early oxygenation measures than intubated thoracic surgery, possibly due to the avoidance of mechanical ventilation-induced barotrauma, volutrauma and atelectrauma [4].

In recent years, supraglottic devices with non-invasive PPV protocols have been employed in nonintubated thoracic surgery as adjuvant tools to treat transient hypoxia or permissive hypercapnia. Moreover, supraglottic devices are also used to deliver positive pressure at the end of each surgical procedure to achieve pulmonary re-expansion [5]. In this respect, even though supraglottic devices have been demonstrated to be less invasive than orotracheal intubation as they avoid tracheal trauma, even when used in spontaneously breathing patients, they still entail the intermittent application of non-invasive PPV, which can be associated with PPV-related lung damage and other complications [6,7]. In particular the forced re-expansion of the lung following PPV may enhance barotrauma, whereas the consequent rapid lung collapse due to restoration of the iatrogenic pneumothorax may lead to endothelial stress failure, capillary leakage and inflammation. 

All these potential adverse effects of PPV can be exaggerated in both emphysematous and interstitiopatic lungs, in which microarchitectural heterogeneity due to areas of poorly ventilated and atelectatic alveoli, and areas with overstretched, hyperinsufflated alveoli, often coexist. 

Conversely, negative-pressure ventilation (NPV) methods more closely mimic physiologic ventilation, and recent data have shown better oxygenation results than PPV, particularly in damaged lungs. This may be the consequence of a more effective ventilation pattern directed toward well perfused areas of the lung in a centripetal rather than centrifugal manner possibly limiting risks of postoperative atelectasis [8,9,10]. 

We thus hypothesized that NPV modes might prove superior to PPV during nonintubated thoracic surgery, particularly in patients with pulmonary emphysema or interstitial lung disease [11,12,13], and that differences between PPV and NPV can be heightened in these patients, eventually affecting their peri-operative outcomes.

In this retrospective study we investigated the effects of an adjuvant transthoracic NPV method compared with adjuvant PPV in two nonintubated thoracic surgery patient cohorts, one with severe emphysema undergoing lung volume reduction surgery (LVRS) and another with undetermined interstitial lung disease undergoing wedge resection for diagnostic purposes. 

## 2. Materials and Methods

This study was designed as a retrospective investigation and included 60 patients operated on via nonintubated thoracic surgery between January 2016 and December 2021 who received adjuvant transthoracic NPV during surgery. Out of these, 30 patients underwent LVRS due to severe emphysema, and another 30 patients underwent lung wedge resection due to undetermined interstitial lung disease. 

The indication for NPV included temporary oxygen saturation < 90%; permissive hypercapnia > 45 mmHg; and the achievement of full lung re-expansion at the end of the procedure. 

Regarding the study aim, the results obtained in the NPV groups were compared to those of a control group of 60 patients selected from a historical cohort via 1:1 propensity score matching analysis and undergoing the same procedures of nonintubated thoracic surgery with non-invasive PPV, applied with the same indications as for NPV. Since Jan 2016, all data were collected in a prospective database according to a standardized protocol. The study was approved by the policlinico Tor Vergata ethical committee (N.11822), and written informed consent for the surgical procedure was obtained from all patients. 

### 2.1. Preoperative Assessment

This assessment included spirometry with plethysmography and diffusion capacity for carbon monoxide (DLCO), assessed using the single-breath technique; arterial oxygen tension (PaO_2_) and arterial carbon dioxide tension (PaCO_2_), assessed via blood gas analysis; and high-resolution chest computed tomography. In both study groups’ patients, indications for surgery were decided following discussion by a multidisciplinary team including pulmonologists, thoracic surgeons and radiologists. 

### 2.2. Inclusion Criteria 

According to the study aim, the main inclusion criteria for LVRS and wedge resection for undetermined interstitial lung disease are presented in Table 1. Patients converted to thoracotomy and orotracheal intubation were excluded. Conversely, conversion to orotracheal intubation due to anesthesiologic complications was included as a secondary outcome.

### 2.3. Anesthesia

Physiological monitoring included venous and radial artery catheterization, electrocardiogram, heart rate, pulse oximetry, assessment of end-tidal CO_2_, blood pressure, blood gases and body temperature. 

Sedation was achieved via an intravenous infusion of propofol plus fentanyl, and the maintenance of spontaneous ventilation was assured via bispectral index monitoring, with values kept between 60 and 90. 

In the PPV group, a laryngeal mask was placed to assist spontaneous ventilation via intermittent PPV in case of temporary SaO_2_ < 90% and end-tidal CO_2_ > 45 mmHg. PPV also was routinely employed to facilitate pulmonary re-expansion at the end of the procedure. 

In the NPV group, a laryngeal mask was placed for additional oxygen delivery, whereas in the case of transient hypoxia with either SaO_2_ < 90% or PCO_2_ > 45 mmHg, NPV was delivered by inserting the surgical suction catheter through the operative access point. The incision was then temporarily closed and made airtight with sterile gauzes placed around the suction catheter, and active aspiration was applied to restore a negative-pressure environment in the pleural cavity. In a similar manner, to achieve pulmonary re-expansion at the end of the surgical procedure, NPV was applied under thoracoscopic vision by connecting the suction catheter to a previously placed chest tube following temporary closure of the surgical incision, as mentioned above. The negative-pressure value was arbitrarily set at a range between −12 and −15 mmHg [14]. In order to achieve this value, the following test was preliminarily conducted before initiation of the study: the surgical aspirator pump was connected to the ventilator through an arterial pressure line, and the system was filled with water. After calipering the 0 value, the aspiration was gradually increased until it reached a value of 12 mmHg, as shown in the ventilator monitor. This corresponded to a value of −75 hPA on the suction system, which was subsequently employed as the standardized negative-pressure value for NPV during the study.

### 2.4. Surgical Technique 

All surgical procedures were performed via video-assisted thoracoscopic surgery through one or two ports. In emphysema patients, LVRS was carried out via a non-resectional staple method in order to plicate the more emphysematous lung regions and reduce the overall lung volume by about 30%. In undetermined interstitial lung disease, staple wedge resection of one or two targeted lung regions, chosen according to both preoperative computed tomography and direct operative lung visualization, was carried for diagnostic purposes. Both surgical techniques have been previously described in detail [15,16].

### 2.5. Postoperative Care 

Blood gas analysis and an assessment of systemic mean artery pressure (MAP) and of the heart rate (HR) were performed at 4 fixed time points: preoperatively (T1; after induction of the surgical pneumothorax (T2); at the completion of the surgical procedure with the patient still sedated (T3); and 60 min after the completion of the surgical procedure (T4). Overall oxygenation is presented as the ratio between PaO_2_ and the fraction of inspired Oxygen (FiO_2_) at the different time points. In addition, the difference (Δ) between the T4 and T1 values of PaO_2_/FiO_2_, PaCO_2_, MAP and HR was calculated. 

Following surgery, all the patients were transferred to a post-anesthesia care unit until the achievement of satisfactory cardio-respiratory parameters, stable vital signs and restored protective reflexes. The indication to discharge the patients from the post-anesthesia care unit to the inpatient ward was decided by the anesthesiology team.

A chest X-ray (CXR) was performed 24 h after the procedure, and the degree of lung expansion was scored as full or incomplete (CXR score of 2 or 1, respectively). The chest tube removal criteria included 24 h drainage < 200 mL with no evidence of air leaks. Postoperatively, all patients underwent early respiratory therapy and the rapid reintroduction of daily activities. Patient discharge was carried out following removal of the chest tube upon achieving stable clinical condition with no complications requiring in-hospital care.

### 2.6. Study Design

This study was designed according to the following background findings: (a) in literature reports, NPV resulted in better oxygenation measures than PPV, particularly in damaged lung [10]; (b) compared to spontaneous ventilation, in patients with impaired pulmonary function, PPV via tracheal intubation and one-lung mechanical ventilation resulted in worse oxygenation 60 min after surgery [3,15,16].

The resulting working hypothesis was that substitution of intermittent noninvasive PPV through a supraglottic device with intermittent transthoracic adjuvant NPV could improve oxygenation 60 min after surgery, particularly in patients with impaired pulmonary function and lung damage either due to severe emphysema or interstitial lung disease.

The primary outcome measure was thus ∆PaO_2_/FiO_2_ at T4. Secondary outcomes included ∆PaCO_2_, ∆MAP, ∆HR, the need for conversion to general anesthesia with intubation, global and per-phase surgical block time, the degree of postoperative pulmonary re-expansion at the CXR, the visual analogue pain score (VAS) at 24 h, operative mortality, morbidity and hospital stay. Global and per-phase surgical block times were defined as follows: anesthesia time was computed as the sum of time needed for the preparation and induction of anesthesia until surgical incision and that needed after skin closure for weaning; operative time was calculated from skin incision to the completion of skin closure; global surgical block time was the overall time spent in the surgical block and was calculated as the sum of anesthesia time, operative time and time spent in the post-anesthetic care unit until discharge to the inpatient ward.

### 2.7. Statistical Analysis

Statistical analysis was conducted using the IBM SPSS Statistical Package (IBM SPSS for Windows, Ver. 25.0, 2017; Armonk, NY, USA). Descriptive statistics were presented as the mean ± the standard deviation. Following a crude analysis of the entire cohort, 1:1 propensity score matching with age, sex, body mass index (BMI), forced expiratory volume in one second (FEV_1_), forced vital capacity (FVC) and DLCO as covariates was performed to create two homogeneous cohorts: an experimental NPV cohort including 30 patients undergoing LVRS and 30 patients undergoing pulmonary wedge for interstitial lung disease, and a control PPV cohort including 2 groups of 30 patients undergoing LVRS and wedge, respectively. 

For each group the standardized mean difference with the range and the 95% confidence interval was calculated, and the homogeneity between the NPV and PPV subgroups was tested via one-way analysis of variance using the same covariates. A comparison between the two groups was performed via one-way analysis of variance with a post-hoc Bonferroni test or analysis of variance for repeated measures with comparisons using the Bonferroni test, and using the chi-square or the Fisher’s exact test (if cells < 5) for frequencies. A *p*-value < 0.05 was considered statistically significant.

## 3. Results

Following propensity score matching, no differences in age, sex, BMI, FEV_1_, FVC or DLCO were detected. The main demographic and preoperative characteristics of the post-matched enrolled population are presented in Table 2.

**Figure 1:**
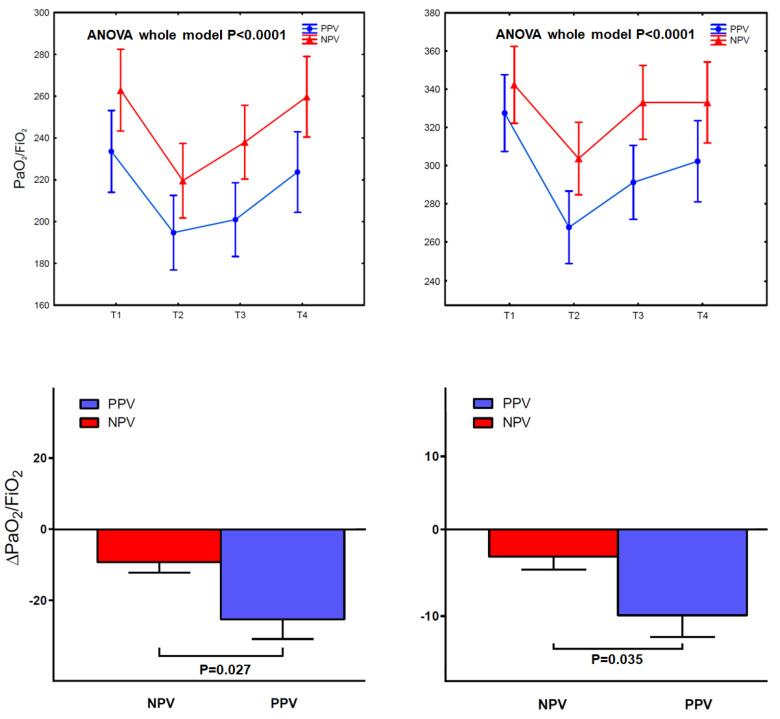
On the left: changes in PaO_2_/FiO_2_ at fixed perioperative periods (up) and comparison with ΔPaO_2_/FiO_2_ (down) in the LVRS group. On the right: changes in PaO_2_/FiO_2_ at fixed perioperative periods (up) and comparison with ΔPaO_2_/FiO_2_ in the wedge group. Vertical bars denote standard error. ANOVA: analysis of variance; FiO_2_: fraction of inspired oxygen; LVRS = lung volume reduction surgery; PaO_2_: arterial oxygen tension; PPV: positive-pressure ventilation; NPV = transthoracic negative-pressure ventilation.

In the enrolled population, NPV could be accomplished in all instances. There were no conversions to open surgery or general anesthesia with intubation. Analysis of the operative times demonstrated that, on average, anesthesia time was 5 min shorter in the LVRS NPV subgroup, whereas the global operative time was 33 min shorter in the wedge NPV subgroups than in the controls, respectively (Table 3). The perioperative changes in PaO_2_/FiO_2_ and ∆PaO_2_/FiO_2_ are depicted in Figure 1. 

**Figure 2:**
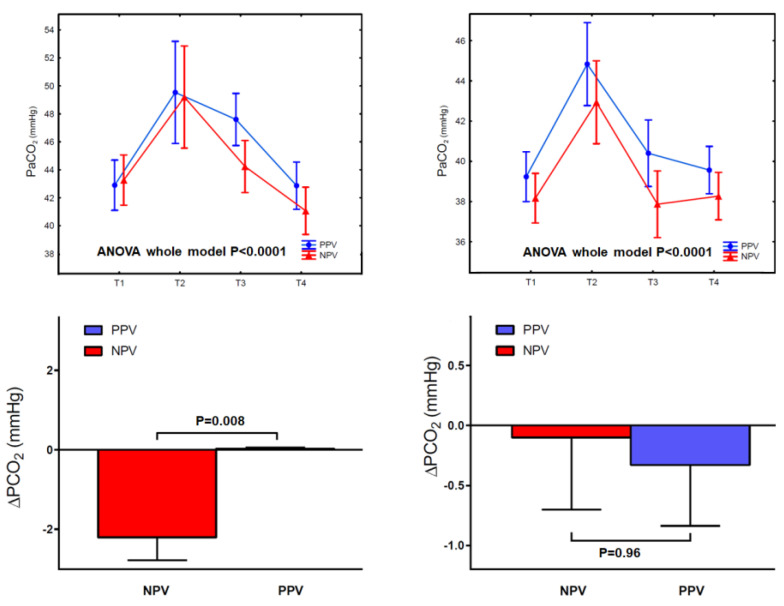
On the left: changes in PaCO_2_ at fixed perioperative periods (up) and comparison with ΔPaCO_2_ (down) in the LVRS group. On the right: changes in PaCO_2_ at fixed perioperative periods (up) and comparison with ΔPaCO_2_ in the wedge group. Vertical bars denote standard error. ANOVA: analysis of variance; LVRS = lung volume reduction surgery; PaCO_2_: arterial carbon dioxide tension; PPV: positive-pressure ventilation; NPV = negative-pressure ventilation.

In both groups, NPV resulted in significant perioperative changes in oxygenation over time with better recovery toward preoperative status at 1 h after surgery. In particular, in the LVRS group, ΔPO_2_/FiO_2_ was lower in the NPV subgroup (9.3 ± 16 mmHg vs. 25.3 ± 30.5 mmHg, *p* = 0.027). Similar results occurred in the wedge group ΔPO_2_/FiO_2_ (3.1 ± 8.2 mmHg vs. 9.9 ± 13.8 mmHg, *p* = 0.035). Amongst the patients undergoing LVRS, PaCO_2_ changed significantly after surgery, with a reduction from pre-operative values of 2.2 ± 3.16 mmHg (*p* = 0.008) at 1 h post-operatively when compared to the PPV group. The differences in PaCO_2_ over time and the absolute ΔPaCO_2_ in both groups are depicted in Figure 2.

**Figure A1:**
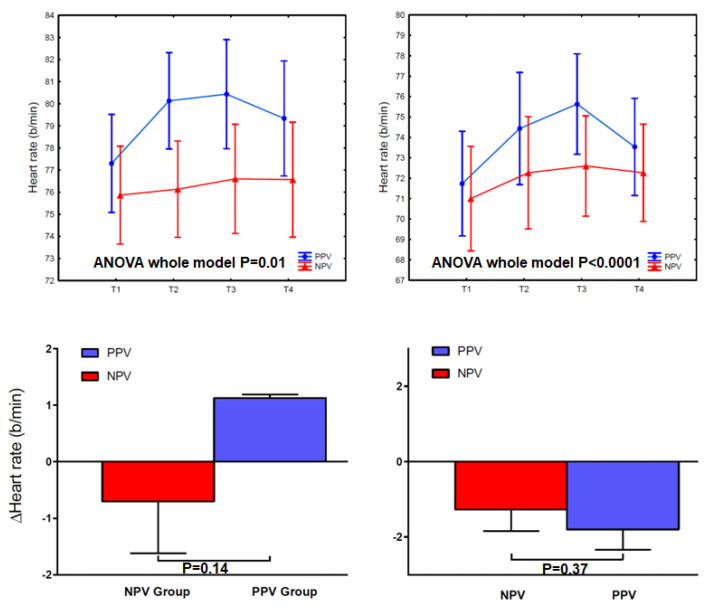
On the left: changes in heart rate at fixed perioperative periods (up) and comparison with Δ heart rate (down) in the LVRS group. On the right: changes in heart rate at fixed perioperative periods (up) and comparison with heart rate in the wedge group. Vertical bars denote standard error. ANOVA: analysis of variance; LVRS = lung volume reduction surgery; PPV: positive-pressure ventilation; NPV = negative-pressure ventilation.

**Figure A2:**
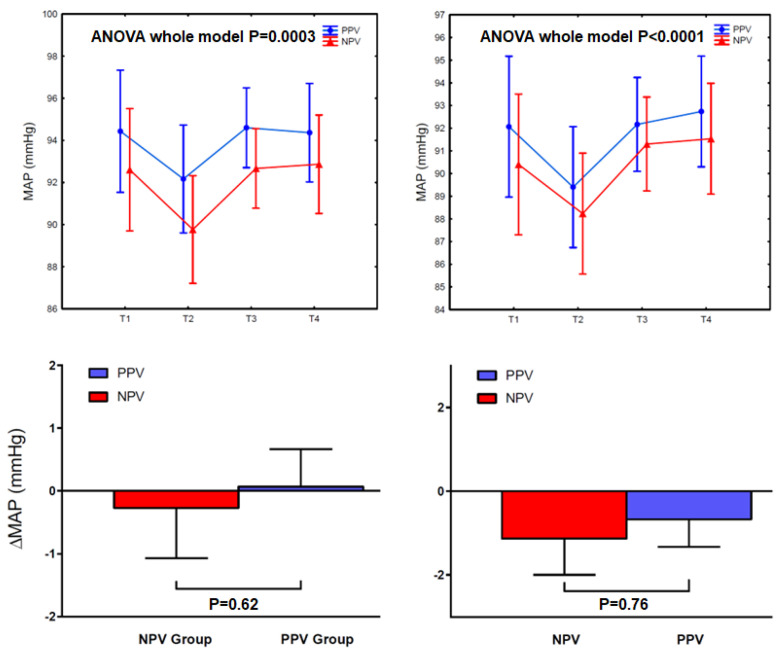
On the left: changes in mean artery pressure at fixed perioperative periods (up) and comparison with Δ mean artery pressure (down) in the LVRS group. On the right, changes in mean artery pressure at fixed perioperative periods (up) and comparison with mean artery pressure in the wedge group. Vertical bars denote standard error. ANOVA: analysis of variance; LVRS = lung volume reduction surgery; PPV: positive-pressure ventilation; NPV = negative-pressure ventilation.

Changes in HR and MAP are depicted in Figure A1 and Figure A2 in Appendix A. There was no operative mortality and no difference in morbidity between the study groups. In the LVRS subgroup, prolonged air leakages were the most frequent post-operative complication, accounting for three out five patients in the PPV and one out of four patients in the NPV group. Other complications in the LVRS included atrial fibrillation, which occurred in two patients in the NPV group and in one in the PPV group; pneumonia in one patient in the PPV group; and subcutaneous emphysema in one patient in the NPV group. In the wedge resection subgroup, prolonged air leakages were detected in three patients, one in the NPV group and two in the PPV group, while one patient in the NPV group developed atrial fibrillation on day 3 following surgery. Other comparative results are shown in Table 3. In particular, in the wedge NPV subgroup, the CXR score was higher than in the controls. No intergroup difference was found in hospital stay.

## 4. Discussion

In this retrospective study, transthoracic NPV was proven safe and effective as an adjuvant ventilation mode in patients with compromised pulmonary function undergoing nonintubated LVRS for severe emphysema and pulmonary wedge resection for undetermined interstitial lung disease. 

In both groups, patients undergoing transthoracic NPV demonstrated better oxygenation measures than the controls 60 min after surgery, as shown by the lower ∆PaO_2_/FiO_2_. In addition, ∆PaCO_2_ was significantly better in the NPV LVRS subgroup, whereas the completeness of lung expansion at the end of the procedure, as indicated by the CXR score, was significantly better in patients undergoing nonintubated wedge resection for the undetermined interstitial lung disease NPV subgroup.

Nonintubated thoracic surgery is continuing to evolve toward minimalistic strategies entailing uniportal surgical access, reducing surgery-related trauma and allowing a progressive transition from more complex regional anesthesia protocols, such as those entailing thoracic epidural anesthesia, toward simpler intercostal block analgesia methods. Similarly, the introduction of a new-generation motorized surgical endoscopic stapler has contributed to minimizing the risk of parenchymal tears during suturing, which may eventually result in prolonged air leak. On the other hand, from an anesthesiologic perspective, while spontaneous ventilation has been maintained as a distinctive element of the nonintubated surgical strategy, the initial “awake” approach has been replaced by the introduction of target-control sedation protocols, including the bispectral index monitoring of levels of consciousness, which now offer optimal patient comfort and improved safety. In this respect, we have found that the overall reduction in surgical and anesthesiologic invasiveness has proven beneficial, particularly in patients with compromised pulmonary function due to severe emphysema or interstitial lung disease [15,16]. 

Despite the maintenance of spontaneous ventilation, noninvasive adjuvant PPV through the laryngeal mask is often employed today during nonintubated thoracic surgery to treat transient hypoxia or permissive hypercapnia and to facilitate re-expansion of the surgically collapsed lung at the end of the procedure [4,15,16]. The adoption of supraglottic devices, such as laryngeal masks, is proven to be well tolerated by patients even without muscular blockage, reducing peri-operative respiratory adverse events when compared to orotracheal intubation [7]. Therefore, in the context of minimized overall surgical and anesthesiologic trauma, supraglottic devices may satisfy the necessity to secure the airways during transient hypoxia or permissive hypercapnia while still preserving the minimal invasiveness strategy. 

The application of NPV through a so called *iron lung* dates back to 1895 [17] and had been historically employed to treat respiratory failure [18]. Though it was considered somewhat obsolete in its original complex construction, the potential usefulness of NPV is still widely debated, and recent reports have shown that it can result in better oxygenation and a greater reduction in lung injury than PPV, particularly in damaged lungs [10,18,19,20]. Our study adds to these findings, showing that NPV applied through a simple, adequately calipered surgical suction catheter, inserted through the surgical access point, resulted in lower post-operative ∆PaO_2_/FiO_2_ in patients with compromised pulmonary function undergoing either nonintubated thoracic surgery LVRS or pulmonary wedge resection for indeterminate interstitial lung disease.

Indeed, during recruiting procedures, including the need for re-expansion of the non-dependent lung via PPV, high pressures and high volumes are delivered to forcedly expand the atelectatic alveoli [21,22]. Although the role of recruiting maneuvers in the development of ventilator-induced lung injury is still debated [23], the effects of high-pressure and/or high-volume ventilation are recognized as causative factors, especially in patients with impaired pulmonary function [13,24] and hyperinflated lung tissue [25]. Within this context, interstitial lung disease can be particularly misleading as the microarchitecture of the interstitiopatic lung is composed of atelectatic alveoli alternating with *ab-extrinseco* overdistended alveoli that are particularly prone to ventilation-related injury [9,10]. The aforementioned physiologic changes are likely to be attenuated but still unsolved in spontaneously breathing patients [4]. Moreover, in nonintubated thoracic surgery, PPV delivered during the recruiting maneuvers may be unmatched with the spontaneous breathing pattern of the patients, leading to patient–ventilator asynchrony, which may also contribute to triggering ventilator-induced lung trauma [6,8,21,22]. 

As far as NPV is concerned, this ventilation mode preferentially drives the ventilatory effort toward a well-perfused area of the lung and is demonstrated to reduce atelectasis when compared with PPV [18,19]. The reasons underlying the advantages of NPV may thus be related to a distribution of pressure over the lung surface, which more closely mimics spontaneous breathing respiratory dynamics. In fact, during spontaneous breathing, NPV was demonstrated to enhance minute ventilation, leading to better oxygenation [26]. These data seem corroborated by our study results and possibly correlate to centripetal rather than centrifugal ventilation of the lung tissue induced by the NPV. Moreover, NPV delivered through this simple and highly reproducible strategy is directed exclusively toward the operated lung, limiting ventilation-related trauma to the dependent lung in which ventilation remains physiologically unaltered. 

### 4.1. Severe Emphysema Subgroup

It is worth noting that in the LVRS group, NPV resulted in lower post-operative PaCO_2_ when compared with the pre-operative values (Figure 2). This result adds to previous findings suggesting that nonintubated thoracic surgery LVRS led to a rapid resolution of permissive hypercapnia 60 min after surgery [14] and that NPV improved gas exchange and reduced dynamic hyperinflation in patients with severe emphysema and respiratory failure [27]. We thus hypothesize that the slight improvement in post-operative PaCO_2_ shown in our study may be due to the cumulative effect achieved, summing up the LVRS effect of reduced dead space ventilation, as well as to the better homogeneity of re-ventilated lung tissue achieved by NPV.

### 4.2. Interstitial Lung Disease Subgroup

Nonintubated thoracic surgery is demonstrated to be an optimal surgical approach to performing diagnostic pulmonary wedge resection in patients with undetermined interstitial lung disease needing a precise pathological diagnosis [28]. 

Indeed, in this particular setting, orotracheal intubation and one-lung mechanical ventilation have been considered major risk factors of post-operative respiratory failure and even death [11,16]. In our study, patients undergoing pulmonary wedge resection with NPV obtained lower ∆PaO_2_/FiO_2_ and a shorter global operating room time when compared to the PPV subgroup. Moreover, patients in the NPV group achieved better pulmonary re-expansion 24 h after surgery than the control PPV group, as shown by their better CXR scores. 

Concerns may be raised due to the risk of NPV-induced pulmonary edema [29]. In order to minimize this risk, in our study protocol, negative pressure was calibrated not to exceed −20 cm H_2_O. This arbitrary value was chosen to keep it far below the range of physiologic negative-pressure swings during maximal inspiratory efforts [14,30]. 

### 4.3. Limitations

This study has some limitations. First, the retrospective nature and the small sample number cannot exclude the risk of bias following the inclusion of non-homogeneous cohorts. Hopefully, this risk was mitigated by the propensity score match, which increased the homogeneity and comparability of the study cohorts. We also expected to find marginal differences in the outcome measures due to the limited temporal application of the two adjunctive ventilation modes during nonintubated thoracic surgery procedures. For this reason, we designed the study to include patients with impaired pulmonary function who were deemed at higher risk for peri-operative oxygenation impairment than other cohorts, a choice that probably allowed us to magnify the benefit of adjuvant NPV in oxygenation measures. The physiologic interpretation of these study results remain speculative. In particular, the unilateral application of a NPV mode may have limited the difference in oxygenation measures when compared to previous experimental studies [18,19]. However, this study was designed to assess differences between nonintubated thoracic surgery ventilation modes, entailing sedation with the maintenance of spontaneous ventilation, plus the on-demand application of PPV through the laryngeal mask, and thus, acting on both lungs (control mode), and sedation with the maintenance of spontaneous ventilation plus the on-demand application of NPV to the non-dependent lung only (experimental mode). 

We thus hypothesized that in patients in lateral decubitus, the application of NPV to the non-dependent lung may have positively affected ventilation in both lungs, firstly by reducing/eliminating rebreathing effects induced by the surgical pneumothorax, and secondly, by reducing the mediastinum shift towards the dependent lung, and thus, eventually increasing its compliance.

## 5. Conclusions

This retrospective study has shown that compared with PPV, nonintubated thoracic surgery with adjuvant transthoracic NPV resulted in better 24 h oxygenation measures in patients with impaired respiratory function undergoing LVRS for severe emphysema or pulmonary wedge resection for interstitial lung disease. NPV also resulted in better CXR scores in patients undergoing pulmonary wedge resection for interstitial lung disease. Further larger prospective studies are warranted to confirm our promising preliminary results.

## Figures and Tables

**Table 1:** Inclusion criteria. RV = residual volume; DLCO = diffusion for carbon monoxide; ASA = American Society of Anesthesiology.

Lung volume reduction surgery for emphysema
Severe heterogeneous emphysema
Severe disability despite maximized medical therapy
RV > 180% predicted
**Pulmonary wedge for interstitial lung disease**
High-resolution computed tomography finding of undetermined interstitial lung disease following multidisciplinary group assessment.
No need for mechanical ventilation
No history of acute exacerbation within the past 6 months
DLCO > 20% predicted
ASA score ≤ 3
Age ≤ 80 years

**Table 2:** Demographics and baseline data of the study groups. LVRS = lung volume reduction surgery; NPV = negative-pressure ventilation; PPV = positive-pressure ventilation; M = male; F = female; BMI = body mass index; FEV_1_ = forced expiratory volume one; FVC = forced vital capacity; TLC = total lung capacity; DLCO = diffusion capacity for carbon monoxide.

	Group LVRS			Group Wedge		
	NPV (*n* = 30)	PPV (*n* = 30)	*p* Value	NPV (*n* = 30)	PPV (*n* = 30)	*p* Value
Age	64.8 ± 8.8	65.0 ± 8.7	0.93	59.6 ± 11	61.9 ± 11	0.32
Sex (M/F)	27/3	25/5	0.66	15/15	18/12	0.51
BMI	22.9 ± 3.4	23.9 ± 3.5	0.40	25.9 ± 2.2	24.9 ± 2.4	0.10
Smoke history	30	29	1.0	16	17	1.0
Comorbidity	11	10	1.0	8	7	1.0
FEV_1_%	27.4 ± 7.0	28.3 ± 9.9	0.9	73.7 ± 12.5	75.2 ± 13.6	0.53
FVC%	67.0 ± 14.0	64.9 ± 13.1	0.59	67.7 ± 12.1	67.6 ± 12.6	0.85
FEV/FVC	0.41 ± 0.10	0.44 ± 0.12	0.54	1.09 ± 0.1	1.13 ± 0.1	0.10
TLC%	127.3 ± 13.0	123 ± 12.6	0.25	76.7 ± 8.4	73.7 ± 7.8	0.08
DLCO%	36.4 ± 5.7	38.5 ± 8.4	0.40	58.1 ± 10.5	59.5 ± 11.9	0.61
Walking test	317 ± 86	327 ± 74	0.84	383 ± 71	370 ± 74	0.55
Dyspnea index	3.30 ± 0.7	3.27 ± 0.7	0.85	1.67 ± 0.7	1.47 ± 0.7	0.28

**Table 3:** Operative results of the study groups. LVRS = lung volume reduction surgery; NPV = negative-pressure ventilation; PPV = positive-pressure ventilation; PO_2_/FiO_2_ = ratio of arterial oxygen tension to fraction of inspired oxygen; PCO_2_ = arterial carbon dioxide tension; SaO_2_ = arterial oxygen saturation; EtCO_2_ = end-tidal CO_2_; T = time; T global surgical block time = sum of anesthesia time, operative time and time spent into the post-anesthetic care unit; VAS = visual analogue pain scale; CXR = chest X-ray.

	Group LVRS			Group Wedge		
	NPV (*n* = 30)	PPV (*n* = 30)	*p* Value	NPV (*n* = 30)	PPV (*n* = 30)	*p* Value
PO_2_/FiO_2_	263 ± 65	233 ± 54	0.08	342 ± 45	321 ± 68	0.26
PCO_2_	43 ± 5.0	42 ± 4.7	0.15	38 ± 3.2	39 ± 3.4	0.24
Temporary SaO_2_ < 90% (N)	3	4	1.0	0	0	1.0
Temporary EtCO_2_ > 45 (N)	14	18	0.43	3	4	1.0
Assisted end-of-procedure lung expansion (N)	30	30	1.0	30	30	1.0
T anesthesia (min)	28 ± 6	33 ± 8	0.01	20 ± 5.7	22 ± 6.0	0.13
T operative (min)	40 ± 11	37 ± 11	0.40	25 ± 6.3	27 ± 7.5	0.19
T global surgical block (min)	145 ± 42	150 ± 43	0.57	86 ± 14	119 ± 31	<0.001
Conversion to intubated anesthesia	0	0	1.0	0	0	1.0
VAS 24 h	2.03 ± 0.9	2.26 ± 0.9	0.29	1.46 ± 0.6	1.63 ± 0.7	0.36
Mortality (N)	0	0	1.0	0	0	1.0
Morbidity (N)	4	5	0.73	2	2	1.0
CXR score	1.80 ± 0.4	1.65 ± 0.5	0.34	1.90 ± 0.3	1.60 ± 0.5	0.04
Hospital stay (days)	6.8 ± 3.9	6.8 ± 5.7	0.77	1.17 ± 0.5	1.26 ± 0.7	0.80

## Data Availability

The data presented in this study are available on request from the corresponding author.
